# Ghrelin and cannabinoids require the ghrelin receptor to affect cellular energy metabolism

**DOI:** 10.1016/j.mce.2012.11.007

**Published:** 2013-01-30

**Authors:** Chung Thong Lim, Blerina Kola, Daniel Feltrin, Diego Perez-Tilve, Matthias H. Tschöp, Ashley B. Grossman, Márta Korbonits

**Affiliations:** a Centre for Endocrinology, William Harvey Research Institute, Barts and the London, London EC1M 6BQ, UK; b Metabolic Disease Institute, Department of Internal Medicine, University of Cincinnati, Cincinnati, OH 45237, USA; c Institute for Diabetes and Obesity, Helmholtz Centre Munich, Department of Medicine, Technische Universität München, Munich, Germany; d Oxford Centre for Diabetes, Endocrinology and Metabolism, University of Oxford, Oxford OX3 7LE, UK

**Keywords:** Ghrelin, Growth hormone secretagogue receptor knockout, Cannabinoids, AMPK, Cannabinoid receptor type 1, Ghrelin receptor

## Abstract

**Introduction:**

Ghrelin is a potent orexigenic brain-gut peptide with lipogenic and diabetogenic effects, possibly mediated by growth hormone secretagogue receptor (GHS-R1a). Cannabinoids also have orexigenic and lipogenic effects. AMPK is a regulator of energy homeostasis and we have previously shown that ghrelin and cannabinoids stimulate hypothalamic AMPK activity while inhibiting it in the liver and adipose tissue, suggesting that AMPK mediates both the central appetite-inducing and peripheral effects of ghrelin and cannabinoids.

**Aims:**

Using GHS-R KO mice, we investigated whether the known ghrelin receptor GHS-R1a is required for the tissue-specific effects of ghrelin on AMPK activity, and if an intact ghrelin signalling pathway is necessary for the effects of cannabinoids on AMPK activity.

**Methods:**

Wild-type and GHS-R KO mice were treated intraperitoneally with ghrelin 500 ng/g bodyweight or CB1 agonist HU210 20 ng/g and hypothalamic, hepatic and adipose AMPK activity was studied using a functional kinase assay.

**Results:**

Ghrelin and HU210 significantly stimulated hypothalamic AMPK activity in wild-type animals (mean ± SEM, 122.5 ± 5.2% and 128 ± 11.6% of control, *p* < 0.05) and inhibited it in liver (55.1 ± 4.8% and 62.2 ± 14.5%, *p* < 0.01) and visceral fat (mesenteric fat (MF): 54.6 ± 16% and 52.0 ± 9.3%, *p* < 0.05; epididymal fat (EF): 47.9 ± 12.1% and 45.6 ± 1.7%, *p* < 0.05). The effects of ghrelin, and interestingly also HU210, on hypothalamic, visceral fat and liver AMPK activity were abolished in the GHS-R KO mice (hypothalamus: 107.9 ± 7.7% and 87.4 ± 13.3%, liver: 100.5 ± 11.6% and 116.7 ± 5.4%, MF: 132.1 ± 29.9% and 107.1 ± 32.7%, EF: 89.8 ± 7.3% and 91.7 ± 18.3%, *p* > 0.05).

**Conclusions:**

Ghrelin requires GHS-R1a for its effect on hypothalamic, liver and adipose tissue AMPK activity. An intact ghrelin signalling pathway is necessary for the effects of cannabinoids on AMPK activity.

## Highlights

► Ghrelin requires GHS-R1a for its effect on hypothalamic, liver and adipose tissue AMPK activity. ► An intact ghrelin signalling pathway is required for the effects of cannabinoids on AMPK activity. ► Ghrelin and cannabinoids stimulate hypothalamic but inhibit liver and visceral fat AMPK. ► Treatment with HU210 at 20 ng/g significantly inhibited inguinal fat AMPK activity. ► We report the loss of inhibitory effects of ghrelin on liver AMPK activity in GHS-R1a KO mice.

## 1 Introduction

Ghrelin is a circulating growth hormone-releasing and appetite inducing brain-gut peptide (Cummings, 2006) that is predominantly synthesised by the endocrine X/A-like cells in the gastric mucosa (Korbonits et al., 2004). It is the endogenous ligand for the previously ‘orphan’ growth hormone secretagogue-receptor (GHS-R), a G protein-coupled receptor with seven transmembrane domains. There are two splicing variants of the GHS-R gene: the functioning full-length product GHS-R1a and a truncated variant GHS-R1b.

One of the most important established roles of ghrelin is the regulation of appetite and energy homeostasis (Kojima et al., 2004; Korbonits et al., 2004). Both central and peripheral administrations of ghrelin cause an increase in appetite and body weight (Kamegai et al., 2001; Tschop et al., 2000; Wren et al., 2001a). Ghrelin acts mainly in the hypothalamus by binding to its receptors in areas that are important for appetite regulation, namely the arcuate nucleus, paraventricular nuclei, dorsomedial region, central nucleus of amygdale and the nucleus of solitary tract (Mano-Otagiri et al., 2006; Olszewski et al., 2003). Ghrelin stimulates the secretion of orexigenic neuropeptide Y, agouti-related protein and orexin, and inhibits the release of anorexigenic compounds such as pro-opiomelanocortin, cocaine- and amphetamine-regulated transcript and α-melanocyte-stimulating hormone, thus resulting in an increase in appetite (Gao and Horvath, 2007; Kohno et al., 2003).

In the liver, ghrelin has opposite effects to insulin on the expression of the enzyme PEPCK, thereby promoting gluconeogenesis and also causing a decrease in glucose uptake and fatty acid oxidation (Rigault et al., 2007). Ghrelin also stimulates hepatic lipogenesis *de novo* in a GH-independent fashion (Sangiao-Alvarellos et al., 2009). Ghrelin promotes adipose tissue deposition by reducing the use of fat as metabolic fuel (Tschop et al., 2000). Intracerebroventricular infusion of ghrelin into neuropeptide-Y (NPY) neurons promotes adiposity, thereby suggesting that the effects of ghrelin on adiposity could be driven through central NPY pathways (Baran et al., 2002). Ghrelin has also recently been shown to play a crucial role in blood glucose maintenance during prolonged extreme hypocaloric conditions via an increase in growth hormone release, and possibly via a direct action (Zhao et al., 2010). All these central and peripheral effects of ghrelin are suggested to be via an effect on the GHS-R.

We have previously shown that ghrelin increases AMP-activated protein kinase (AMPK) activity in the hypothalamus and inhibits it in the liver and adipose tissue (Kola et al., 2005), suggesting that AMPK mediates at least some of the central and peripheral metabolic effects of ghrelin. AMPK is a key enzyme regulator in energy homeostasis both at cellular and whole body energy levels (Kola et al., 2006; Xue and Kahn, 2006). Activated AMPK inhibits anabolic pathways as well as stimulating catabolic pathways and appetite, thus conserving the ATP levels (Xue and Kahn, 2006).

Cannabinoids are also orexigenic compounds that have been shown to increase food intake (Williams et al., 1998) via the cannabinoid (CB)-1 receptor (Jamshidi and Taylor, 2001; Williams and Kirkham, 1999). Peripherally, cannabinoids promote lipogenesis in the liver (Osei-Hyiaman et al., 2005). We have also shown that cannabinoids increase AMPK activity in the hypothalamus and inhibit it in the liver and adipose tissue (Kola et al., 2005).

It has been shown that the effects of ghrelin on food intake and AMPK activity are absent in CB1 knock-out (KO) or CB1 receptor antagonist rimonabant-treated rodents (Kola et al., 2008). Ghrelin also increased the hypothalamic endocannabinoid content in wild-type (WT) mice, but not in CB1 KO or rimonabant-treated mice (Kola et al., 2008). These results suggested that an intact cannabinoids signalling system is necessary for the effects of ghrelin on AMPK activity.

By using GHS-R KO mice model, we aimed to investigate whether the GHS-R is required for the effects of ghrelin on AMPK activity of different tissues, and if an intact ghrelin signalling pathway is necessary for the effects of cannabinoids on AMPK activity. We hypothesised that ghrelin requires the presence of GHS-R for its effects on AMPK activity of different tissues. We also hypothesised that the effects of cannabinoids on AMPK activity of different tissues require the presence of an intact ghrelin–GHS-R signalling pathway.

## 2 Materials and methods

### 2.1 In vivo study

Male WT C57BL/6 J mice and GHS-R KO mice (Sun et al., 2004) weighing approximately 35 g and age-matched were used in the study, each equally divided into three groups. Animals were housed individually under standard environmental conditions (light from 6 AM to 6 PM, temperature 22 ± 1 °C). The mice were handled regularly and were given intraperitoneal (ip) injection for 4 days before the study day with saline or the CB1 agonist HU210 (2, 5 or 20 ng/g, Tocris Bioscience), to get them accustomed to injection and to the cannabinoid effect, as suggested previously (Kirkham and Tucci, 2006). The average food intake of chow diet after 1, 2, 3 and 4 h post-injection were measured on the day before the study day.

The mice were again fed *ad libitum* the night before. On the day of Experiment 2, they were treated with vehicle (saline), ghrelin 500 ng/g or HU210 20 ng/g. The doses were chosen for their established effectiveness in earlier studies to promote increases in food intake (Kirkham et al., 2002; Osei-Hyiaman et al., 2005). Food was removed immediately upon treatment, leaving them with water only. One hour later the mice were killed and the hypothalamus, liver, fat pads (inguinal, mesenteric and epididymal fat: IF, MF and EF respectively) and pituitary gland were removed. Tissue samples were frozen in liquid nitrogen and stored at −80 °C directly. The experimental procedures carried out in this study were in compliance with the UK Animals (Scientific Procedures) Act 1986.

### 2.2 GHS-R and CB1 receptors expression study

To confirm the absence of GHS-R expression in GHS-R KO mice and to study the expression of CB1 receptor in both WT and GHS-R KO mice, RNA was extracted from the pituitary glands of WT and GHS-R KO mice using Qiagen RNA extraction kit. RNA was quantified using Nanodrop spectrophotometer and RT-PCR was performed to obtain cDNA. Each reaction contained 1 μg of DNase I-treated total RNA isolated from different mouse tissues and primers for GHS-R1a (forward primer 5′ GTATGGGTGTCGAGCGTCTT 3′, reverse primer 5′ CTTCTGGTGTGGAGCAATGA 3′) or CB1 receptor (forward primer 5′ CTGGTTCTGATCCTGGTGGT 3′, reverse primer 5′ TGTCTCAGGTCCTTGCTCCT 3′) respectively. The cycling parameters for 35 cycles were set as 94 °C, 30 s; 60 °C, 1 min; and 72 °C, 30 s. Samples were loaded onto 2% agarose gel.

### 2.3 AMPK activity assay

The kinase assay for AMPK activity has been described previously (Hawley et al., 2003; Kola et al., 2005). Briefly, the tissues of interest (hypothalamus, fat pads and liver) were weighed and homogenised with Precellys 24 using CK14 tubes containing ceramic beads (Stretton Scientific, Stretton, UK) at 6000 rpm for 1 cycle of 20 s in tissue lysis buffer containing 50 mM Tris–HCl, 50nM NaF, 5 mM Na pyrophosphate, 1 mM EDTA, 10% (v/v) glycerol, 1% Triton X-100, 1 mM dithiothreitol (DTT), 1 mM benzamidine, 1 mM phenylmethane sulfonyl fluoride (PMSF), 5 μg/ml soybean trypsin inhibitor (SBTI) and protease inhibitor (for hypothalamus, hypothalamic lysis buffer containing 50 mM Tris–HCl, 50 mM NaF, 5 mM Na pyrophosphate, 1 mM EDTA, 250 mM sucrose, 1% Triton X-100, 1 mM DTT, 1 mM benzamidine, 0.1 mM phenylmethane sulfonyl fluoride, 5 mg/ml soybean trypsin inhibitor and protease inhibitor was used). Protein content was determined using BCA assay (Pierce, Rockford, USA). AMPK was immunoprecipitated with an equal mixture of α1-AMPK and α2-AMPK antibodies (Hawley et al., 2003) and AMPK activity was determined by the entity of phosphorylation of SAMS (Pepceuticals Ltd., Nottingham, UK), a synthetic peptide substrate of AMPK.

### 2.4 Statistical analysis

Data were analysed using the ANOVA followed by the Newman–Keuls test or the Kruskal–Wallis test followed by Conover–Inman comparison, as appropriate. *p* < 0.05 was considered significant and data were expressed as means ± standard error in each treatment group.

## 3 Results

### 3.1 GHS-R and CB1 receptors expression study

**Fig. 1:**
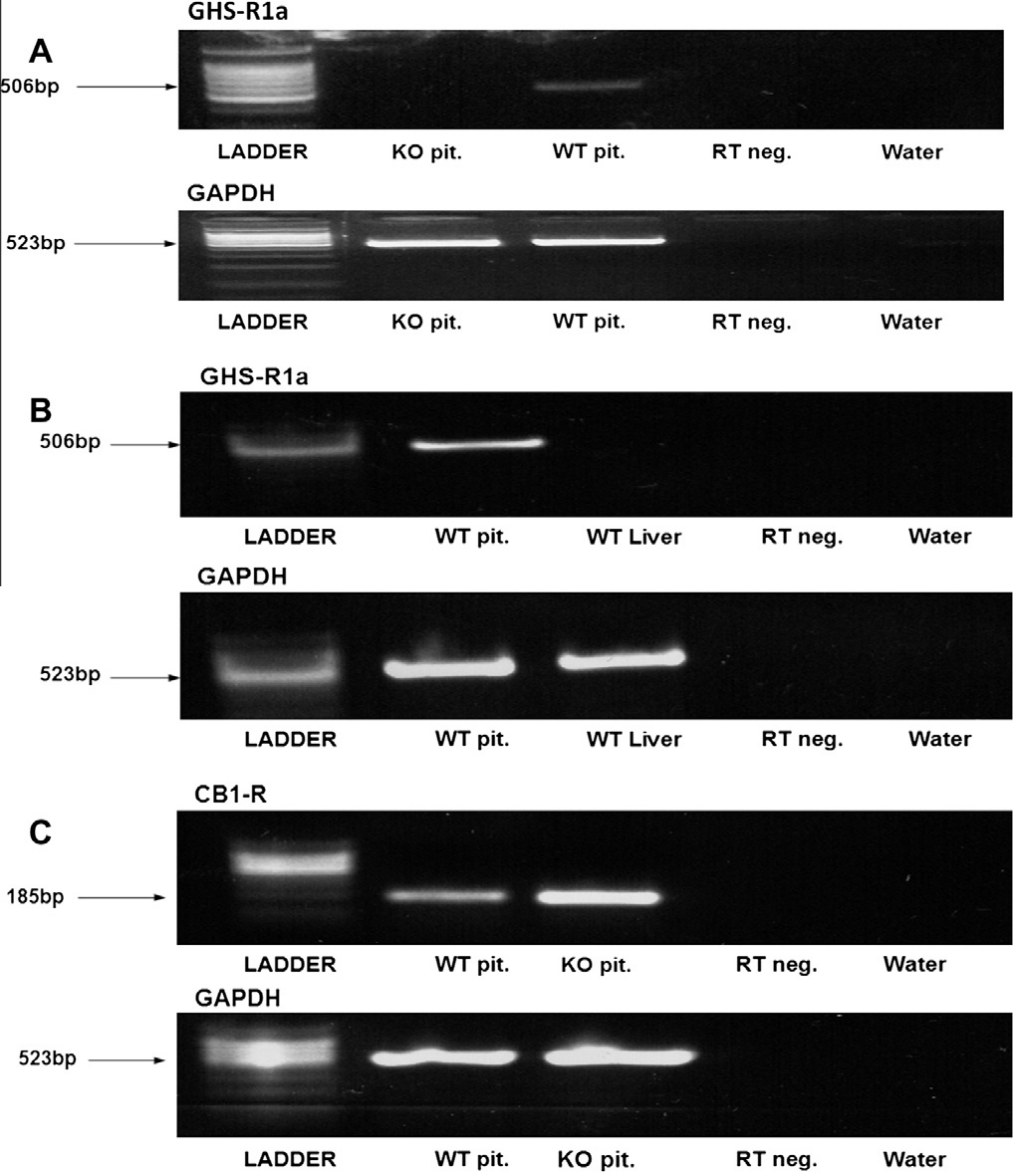
(A) RT-PCR using the pituitary tissues to confirm that the GHS-R KO mice were not expressing the functional GHS-R1a. (B) RT-PCR showing the absence of GHS-R1a expression in the liver of WT mice. (C) RT-PCR showing the normal expression of CB1 receptor in the pituitary of GHS-R KO mice.

The GHS-R KO mice were confirmed to show no expression of the functional GHS-R1a (Fig. 1A). GHS-R1a was also not expressed in the liver of WT mice (Fig. 1B), as previously shown (Gnanapavan et al., 2002). The GHS-R KO mice had a normal expression of the CB1 receptor, as shown by RT-PCR (Fig. 1C).

### 3.2 Food intake

**Fig. 2:**
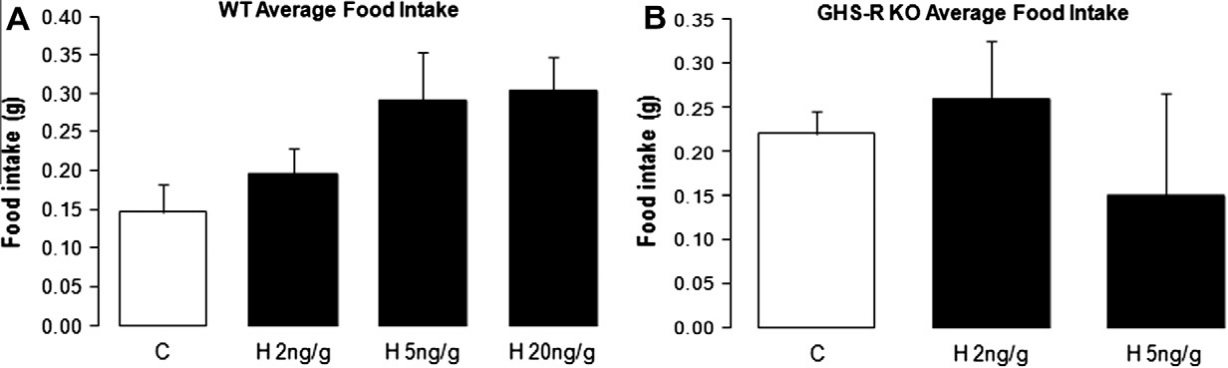
The effects of different doses of HU210 injection on average food intake of chow diet measured 4 h post-injection in WT (A) and GHS-R KO (B) mice. The results are shown as mean food intake ±SEM g/h (C, control; H, HU210).

The data on mean food intake at 4 h after HU210 injection suggest a trend in response to increasing doses of CB1 agonist HU210 in WT mice (Control (C): 0.15 ± 0.03 g/h; HU210 (H) 2 ng/g: 0.20 ± 0.03 g/h; H 5 ng/g: 0.29 ± 0.06 g/h; H 20 ng/g: 0.30 ± 0.04 g/h; *p* = 0.15, Fig. 2A); however, the differences did not reach significance. Food intake in response to HU210 showed no significant difference in GHS-R KO mice compared to control (C: 0.22 ± 0.03 g/h; H 2 ng/g: 0.26 ± 0.06 g/h; H 5 ng/g: 0.15 ± 0.12 g/h; *p* = 0.11, Fig. 2B).

### 3.3 AMPK activity

#### 3.3.1 Hypothalamus

**Fig. 3:**
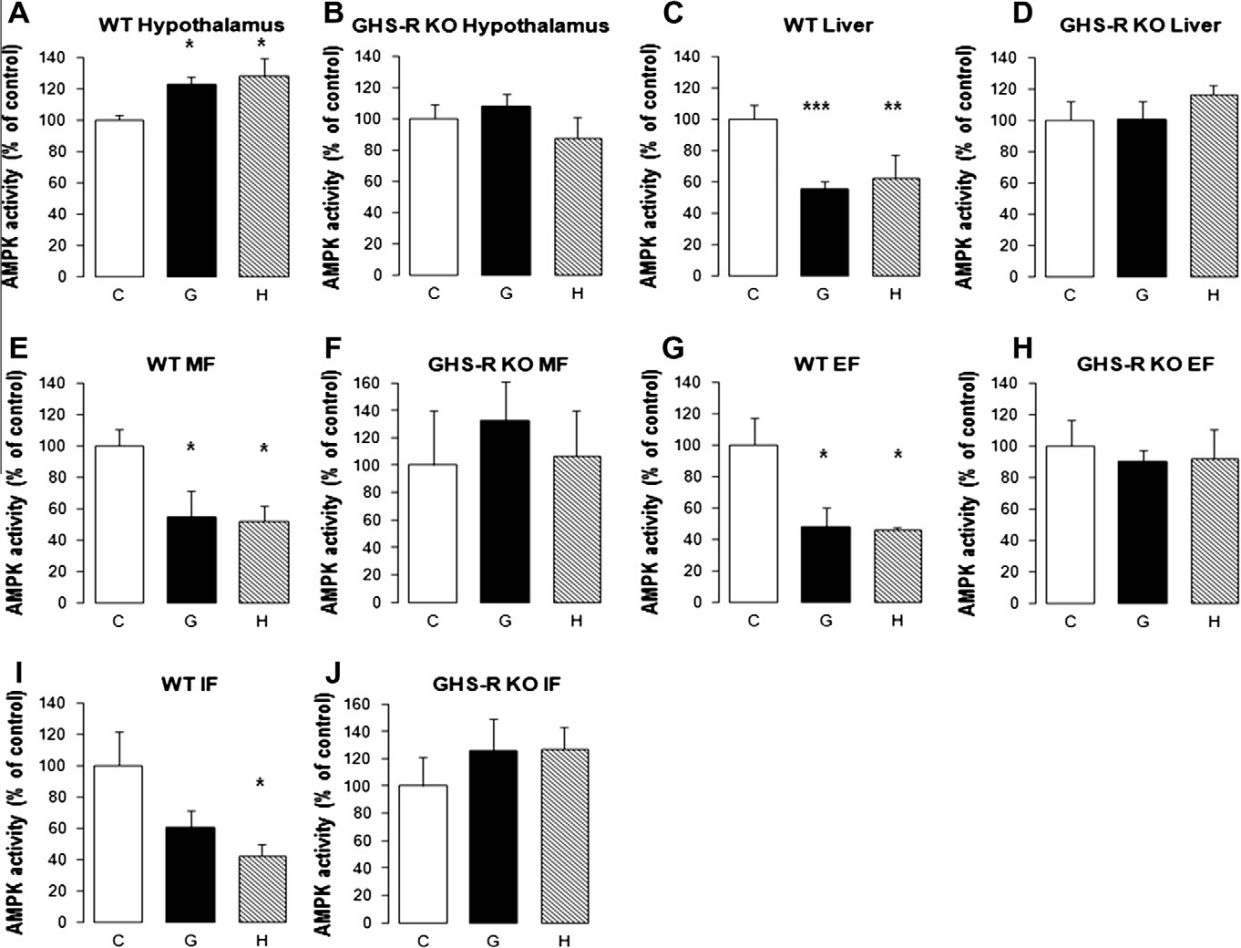
The effects of ghrelin and cannabinoids on AMPK activity of different tissues in WT and GHS-R KO mice. 3A and 3B: Ghrelin and cannabinoids effects on WT and GHS-R KO hypothalamic AMPK activity 1 hour after intraperitoneal administration of vehicle (C), ghrelin 500 ng/g (G) or HU210 20 ng/g (H); 3C and 3D: Ghrelin and cannabinoids effects on WT and GHS-R KO liver AMPK activity; 3E and 3F: Ghrelin and cannabinoids effects on WT and GHS-R KO mesenteric fat AMPK activity; 3G and 3H: Ghrelin and cannabinoids effects on WT and GHS-R KO epididymal fat AMPK activity; 3I and 3J: Ghrelin and cannabinoids effects on WT and GHS-R KO inguinal fat AMPK activity. (WT: *n* = 7/treatment group; GHS-R KO: *n* = 6/treatment group; ^*^: *p* < 0.05; ^**^: *p* < 0.01; ^***^: *p* < 0.005) (C, control; G, ghrelin; H, HU210).

Both ghrelin and HU210 significantly stimulated hypothalamic AMPK activity in WT mice (ghrelin: 122.5 ± 5.2% of control, *p* < 0.05; HU210: 128.0 ± 11.6% of control, *p* < 0.05; Fig. 3A). In GHS-R KO mice, the stimulatory effects of ghrelin and HU210 on hypothalamic AMPK activity were lost (ghrelin: 107.9 ± 7.7% of control, *p* = 0.4579; HU210: 87.4 ± 13.3% of control, *p* = 0.6197; Fig. 3B).

#### 3.3.2 Liver

Both ghrelin and HU210 significantly inhibited AMPK activity in the liver of WT mice (ghrelin: 55.1 ± 4.8% of control, *p* < 0.01; HU210: 62.2 ± 14.5% of control, *p* < 0.01; Fig. 3C). The inhibitory effects of ghrelin and HU210 on liver AMPK activity were lost in GHS-R KO mice (ghrelin: 100.5 ± 11.6% of control, *p* = 0.8354; HU210: 116.7 ± 5.4% of control, *p* = 0.307; Fig. 3D).

#### 3.3.3 Visceral fat

Both ghrelin and HU210 significantly inhibited AMPK activity in MF of WT mice (ghrelin: 54.6 ± 16.6% of control, *p* < 0.01; HU210: 52.0 ± 9.3% of control, *p* < 0.01; Fig. 3E). The inhibitory effects of ghrelin and HU210 on MF AMPK activity were lost in GHS-R KO mice (ghrelin: 132.1 ± 29.9% of control, *p* = 0.5459; HU210: 107.1 ± 32.7% of control, *p* = 0.8793; Fig. 3F).

Similarly, ghrelin and HU210 also significantly inhibited AMPK activity in EF of WT mice (ghrelin: 47.9 ± 12.1% of control, *p* < 0.05; HU210: 45.6 ± 1.7% of control, *p* < 0.05; Fig. 3G). Again, the inhibitory effects of ghrelin and HU210 on EF AMPK activity were abolished in GHS-R KO mice (ghrelin: 89.8 ± 7.3% of control, *p* = 0.632; HU210: 91.7 ± 18.3% of control, *p* = 0.5111; Fig. 3H).

#### 3.3.4 Subcutaneous fat

Ghrelin showed a slight but non-significant inhibitory effect on IF AMPK activity in WT mice (60.6 ± 10.2% of control, *p* = 0.2132; Fig. 3I). In contrast, HU210 significantly inhibited IF AMPK activity in WT mice (41.9 ± 7.7% of control, *p* < 0.05; Fig. 3I). In GHS-R KO mice, both ghrelin and HU210 showed no effect on AMPK activity in IF (ghrelin: 125.9 ± 22.8% of control, *p* = 0.3881; HU210: 127.0 ± 16.1% of control, *p* = 0.3504; Fig. 3J).

## 4 Discussion

AMPK is a highly conserved serine/threonine kinase which is identified as one of the key players in the regulation of appetite and metabolism (Minokoshi et al., 2004). Several upstream regulators of AMPK have been identified (Lim et al., 2010), and AMPK is also known to mediate the effects of some metabolic hormones and elements (Lim et al., 2010). In general, activated AMPK will lead to the stimulation of appetite and activation of catabolic processes such as fatty acid oxidation, glycolysis, increased glucose uptake and mitochondrial biogenesis (Lim et al., 2010). Inhibition of AMPK activity conversely leads to hepatic gluconeogenesis, lipogenesis, and glycogen synthesis (Lim et al., 2010).

Acute injections of ghrelin and cannabinoids have been shown to increase appetite in rats (Kirkham, 2003; Wren et al., 2001b) and mice (Avraham et al., 2004; Kola et al., 2008; Sun et al., 2004; Wiley et al., 2005). HU210 is a highly potent synthetic cannabinoid receptor agonist (*K_i_* values of 0.061 and 0.52 nM at cloned human CB1 and CB2 receptors respectively – data from Tocris Bioscience). In this study, we attempted to investigate the effects of HU210 injection on food intake (Fig. 2A and B). It is known that food intake in mice is more difficult to measure than rat models (Graham Tobin and Russell, 2007). Food intake in control mice showed a trend to increase with higher doses of HU210 injection (Fig. 2A) and no significant difference in food intake was found in the GHS-R KO mice (Fig. 2B). We speculated that the presence of GHS-R1a may be required for the orexigenic effects of cannabinoids. A future study investigating the effects of a CB1 agonist on food intake in the presence of a ghrelin receptor antagonist will be useful.

Previously, we have shown that ghrelin and cannabinoids stimulate hypothalamic AMPK activity (Kola et al., 2005). In contrast, both the orexigenic compounds have inhibitory effects on AMPK activity in the liver and visceral fat (Kola et al., 2005). In this study, we were able to reproduce our previous results. Both ghrelin and HU210 administration had a stimulatory effect on WT hypothalamic AMPK activity. Similarly, both led to a significant reduction of AMPK activity in WT liver and visceral fat. Previous studies have shown that ghrelin and cannabinoids have no effect on AMPK activity of subcutaneous fat (Kola et al., 2005). Here, we show that the effect of ghrelin on WT IF (equivalent to subcutaneous fat) AMPK activity was comparable to controls (*p* = 0.2132). However, treatment with HU210 at 20 ng/g significantly inhibited WT IF AMPK activity (*p* = 0.0252). The high potency of HU210 might explain the lack of a significant effect in previous studies using the less potent THC, AEA, and 2-AG (Kola et al., 2005).

Consistent with a previous report (Andrews et al., 2008), the stimulatory effect of ghrelin on hypothalamic AMPK activity was diminished in GHS-R KO mice. Here, we show that ghrelin administration reduces AMPK activity in peripheral tissues such as liver and visceral fat AMPK, an effect that also requires the expression of GHS-R. This is in line with previous data which have shown that GHS-R mediates the orexigenic effects of ghrelin, and a GHS-R antagonist was able to reduce food intake and to block the effects of ghrelin on adipose tissue (Asakawa et al., 2003).

Previous studies have shown that the liver only expresses the non-functional GHS-R1b, and GHS-R1a is known to be absent at this site (Gnanapavan et al., 2002). We confirmed that GHS-R1a expression is absent in the liver of WT mice (Fig. 2). Thus, the loss of inhibitory effect of ghrelin on the liver AMPK activity in GHS-R KO mice suggests that this effect is mediated by GHS-R1a activity in other tissues. Studies involving the use of mouse models with tissue-specific expression of GHS-R may be useful in exploring the mechanisms whereby ghrelin inhibits liver AMPK activity.

We show here that the stimulatory effect of HU210 on hypothalamic AMPK activity, as well as its inhibitory effect on AMPK activity on liver and subcutaneous and visceral fat, requires the expression of GHS-R. Considering the key role of AMPK activity in the control of energy metabolism, this result suggests that ghrelin signalling is an essential component of the pathways by which cannabinoids control energy balance. Interestingly, there is evidence that the orexigenic effect of ghrelin requires a functional endogenous cannabinoid system. Hence, administration of the CB1 receptor antagonist rimonabant blunted the orexigenic effect of ghrelin (Tucci et al., 2004). Furthermore, ghrelin failed to increase feeding in CB1-KO mice (Kola et al., 2008), suggesting that at least CB1 receptor is mandatory for the orexigenic effects of ghrelin and cannabinoids and their influence on AMPK activity. In addition, ghrelin increased the cannabinoid content of the hypothalamus, and this effect was CB1-dependent (Kola et al., 2008). Rimonabant also blocked the stimulatory effects of ghrelin on 2-AG content (Kola et al., 2008) and abolished the inhibitory effects of ghrelin on the excitatory synaptic input in the PVN (Kola et al., 2008). These studies have substantiated the involvement of cannabinoids system in the effects of ghrelin.

In this study, we propose that an intact ghrelin pathway is also required for the tissue-specific AMPK effects of cannabinoids. The effects of HU210 on AMPK activity were totally abolished in the hypothalamus, liver, subcutaneous and visceral fat of GHS-R KO mice. Our data indicate:

(i) The control of AMPK activity by cannabinoids requires an intact ghrelin signalling pathway.
(ii) An interaction between GHS-R and CB1 receptor may be necessary for the effects of cannabinoid–CB1 binding complex and downstream effects. The interaction might be in the form of heterodimerisation. Although G protein-coupled receptor dimerisation is a relatively novel concept, both GHS-R and CB1 have shown to dimerise with other partners: formation of GHS-R/dopamine-R1 heterodimers (Jiang et al., 2006) and CB1/Angiotensin-R1 heterodimers (Turu et al., 2007) have been published. Heterodimerisation of GHS-R and CB1 has yet to be shown in any studies, and there is always a possibility that cannabinoids require the presence of GHS-R/CB1 heterodimer for their orexigenic and tissue-specific AMPK effect.

In conclusion, we confirmed in this study that ghrelin requires the presence of GHS-R for its effects on AMPK activity in the hypothalamus, liver and visceral fat. We also showed that the effects of cannabinoids on hypothalamic, liver, visceral and subcutaneous fat AMPK activity require the presence of GHS-R, therefore suggesting that an intact ghrelin signalling pathway is necessary for these effects of cannabinoids.
